# Professional Ethics

**Published:** 1873-08

**Authors:** G. P. Holmes

**Affiliations:** Lawrence, Kansas


### DENTAL REGISTER.

Vol. XXVII.] AUGUST, 1873. [No. 8.

### PROFESSIONAL ETHICS.

BY G. P. HOLMES, LAWRENCE, KANSAS.

The subject of professional ethics should receive the careful consideration of every dentist. It has much to do with his success or failure in life. Let him enter society with a refined polished manner, an amiable desire to please, and he will be met in a like spirit. Extend a friendly hand of greeting, and kindred greetings meet you on every side. On the contrary, rude manners, harsh words, sharp thrusts will be met with resistance and sharp retorts. The dentist especially has need of these accomplishments. Society and his profession expect much of a professional man, and his success is made easier by their possession. He depends upon the patronage of the public, then let him add to his professional skill, then when if possessed of excellent qualities, the requisite amount of energy, his road to success will be found easy.

The dentist owes a duty to his profession; to it he must be either an ornament or a disgrace. The profession has a right to demand that all its members shall be somebody, that their works shall show them to be *worthy members.* The Drs., M. D. and D. D. S., upon cards and signs are but little guarantee of ability. Society has so often been cheated by them, that he who flourishes those titles most, ever making them conspicuous, is looked upon with distrust. They are no mark of brains, their profession, or rather the knowledge which their possessor is supposed to have, is highly important. They should only be regarded as an evidence that the object had been sought for—not as the end sought. Let every one prove his ability, and his manhood, and society will extend to him its friendship and its patronage.

Were all alike possessed of a desire to deal fairly and honorably, having a proper appreciation of our profession in view, there would be little need of a Code of Ethics. That some acknowledged standard is desirable, I think none will question. The highest and most reputable organization in our country have so thought and acted, and to-day the Code of Ethics of the American Dental Association is adopted by most local and state organizations.

The primary object of these organizations is to disseminate knowledge, and elevate the standard of qualifications. If so, then whatever tends to retard dental progress, should be com batted, discountenanced and removed through their influence. It is highly important that a dental association should set its standard high, that it should require that all who become members shall be possessed of a good moral character, shall occupy an honorable position in society—where they are best known, and that if they are not well qualified they shall be earnest seekers after knowledge in the profession.

The Dentist should be a well bred gentleman, affable and kind, gentle though firm in his dealings with his patients. He is called upon to inflict severe pain, let him encourage his patients with a kind word, show them that whilst he inflicts and makes them suffer, he is not devoid ot feeling.

The Dentist should be clean. The qualityof his clothes is of minor consideration, but it is important that he be clean, with him especially is cleanliness a virtue, which he cannot afford to be without.

His office should be orderly, his instruments, chair and sur roundings, must receive daily attention. It would seem that these were so plainly and evidently necessary, that a reference to them would be unnecessary, but many neglect them to the disgust of their patients, and to their own detriment. Those who have students under their charge neglect a most important duty, if they fail in teaching the necessity of cleanliness.

As members of the dental profession we are bound to maintain its honor, and labor to extend its usefulness. He that will not or does not use his influence to promote the interests of his chosen profession is recreant to his duty. He who sits supinely down in his office and does nothing aside from his daily routine of practice, or who becomes so engrossed with business as to leave no time for improvement, is a dead weight to be carried along by the energy of other members. Others there are who are so possessed of self-conceit as to close all avenues of improvement—who look upon dental associations as bores, and upon their regulations with contempt. There may still be hope for them, for after years of experience they may be brought to comprehend how little they know, and learn of their own insignificance. Still they are to be classed among the dead weights to be borne along by others if at all in the onward march of improvement.

The subject of dental advertising demands attention. Among all the list of causes none tends more to hinder our progress than the custom of advertising, calling attention to the wonderful skill, the acquired fame, the unsurpassed facilities, of the undersigned, who is perhaps a graduate, or has practiced successfully for a long time in other parts, to produce *first-class* and at the same time the cheapest work in the town! This style of advertising was once excusable—many of us no doubt can look back, without pride, upon some of our own productions of this kind. Our education was at fault. There is no longer excuse. That form of advertising hasn't an advocate in all our literature. Thanks to associated effort, to dental literature, and dental colleges. It should receive the most public disapproval of all our associations, and through the influence of its members be driven from among us.

In a report presented at the last meeting of the State Association the Committee say, "That after careful investigation, reading and comparing hand-bills, posters, newspaper advertisements and show cases, they had come to a stand still—hardly knowing whether we represented a profession or a trade—but judging from the matter investigated they were almost led to the belief that it was all "bosh" about "Profession" and "professional standing." How long these practices shall continue depends in a great measure upon us. Let us adopt some standard, that in our practice and teaching we will tend to elevate our vocation to a place among the learned professions.

The code of ethics of the American Dental Association is a high-toned professional production. It is calculated to expand and elevate the mind of the dentist, to point out plainly and forcibly his duties to himself, his patrons, and the profession. Many of the unprofessional customs, which are so well known to you as belonging to men of our profession in Michigan, and even in this Association, are there properly re-rebuked. The line of duty is in many ways clearly pointed out to the practitioner. With justice and dignity it effectually negatives many a mistaken notion of professional ethics.

